# Evaluation of urinary biomarkers for detection of renal tubular injury in *Vipera berus-*envenomated and critically ill dogs with and without clinically apparent acute kidney injury

**DOI:** 10.1186/s12917-026-05939-1

**Published:** 2026-09-21

**Authors:** Anna K. Selin, Inger Lilliehöök, Emma M. Strage, Anders Larsson, Lena Pelander

**Affiliations:** 1 Department of Clinical Sciences, Swedish University of Agricultural Sciences, Uppsala, Sweden; 2 AniCura Albano Animal Hospital, Stockholm, Sweden; 3 Department of Medical Sciences, Clinical Chemistry, Uppsala University, Uppsala, Sweden

**Keywords:** Canine, Tubular, AKI, *N*-acetyl-β-D-glucosaminidase, Cystatin C, Glucose

## Abstract

**Background:**

Acute kidney injury (AKI) in dogs has high mortality and early diagnosis is essential, but traditional markers are not sensitive enough to detect renal injury in the absence of decreased glomerular filtration rate. The objective of this study was to compare urinary cystatin C (uCysC), *N*-acetyl-β-D-glucosaminidase (uNAG), and glucose (uGlu) among dogs hospitalised because of envenomation by *Vipera berus* (EVB) or admitted for intensive care without (IC) or with (AKI_APP_) clinically apparent AKI, and 30 healthy dogs. Urine and serum samples were collected on days 1 and 2, and urine samples on days 7 and 14. Urinary CysC, NAG, and glucose concentrations were measured with an automated chemistry analyser, normalised to urinary creatinine concentration (uCr) and compared among groups.

**Results:**

Ninety-seven dogs were included (30 HC, 28 EVB, 14 AKI_APP_ and 25 IC dogs). Age, bodyweight, and sex did not differ among groups. On day 1, median [IQR] uCysC/uCr (10^–3^) and uGlu/uCr were higher (*p* < 0.008) in EVB (0.05 [0.03–0.11], 0.04 [0.03–0.06)]), IC (0.06 [0.03–0.4], 0.05 [0.03–0.09]), and AKI_APP_ (4.4 [0.13–30.0], 0.12 [0.05–0.45]) groups, compared to healthy dogs (0.02 [0.01–0.03], 0.03 [0.02–0.04]). Urinary markers decreased over two weeks post hospitalisation in all groups except AKI_APP_. Initial levels of uCysC/uCr were higher (*p* < 0.002) and remained elevated in non-survivors compared to survivors of AKI_APP_, suggesting potential prognostic value.

**Conclusions:**

Urinary CysC/uCr and Glu/uCr are potential markers of tubular injury in early stages of AKI in dogs.

**Supplementary Information:**

The online version contains supplementary material available at https://doi.org/10.1186/s12917-026-05939-1.

## Introduction

Acute kidney injury (AKI) in dogs is relatively common and associated with high mortality [1–6]. Early diagnosis might improve survival by enabling treatment before irreversible renal injury and uremia occur [7, 8]. Urinary biomarkers have the potential to identify renal injury or dysfunction earlier than conventional serum markers (creatinine, SDMA), which detect decreased glomerular filtration rate (GFR) [9]. Some urinary markers may also localise renal injury, for example detection of glomerular injury by urinary IgG and CRP [9]. Sensitive and specific markers for early detection of tubular injury are needed in the clinical setting.

Cystatin C (CysC) is a 13 kDa cysteine protease inhibitor produced by all nucleated cells, freely filtered by glomeruli and reabsorbed by proximal tubular epithelial cells [10, 11]. Urinary cystatin C (uCysC) increases with proximal tubular injury [12, 13]. In experimental AKI in dogs uCysC increases before azotaemia, and histological evidence of renal tubular injury correlates with uCysC in dogs with gentamicin or tenofovir-induced AKI [14–16]. The value of urinary enzymes for detection of renal tubular injury has been investigated previously [16–19]. *N*-acetyl-β-D-glucosaminidase (NAG) is an enzyme released with renal tubular injury. Earlier studies in dogs suggest that urinary NAG (uNAG) can be used in toxicity testing due to its sensitivity for detection of renal tissue injury [16, 20], though few studies exist on uNAG in naturally occurring AKI in dogs [21, 22].

Glucose can increase in urine because of impaired proximal tubular reabsorption [23]. In contrast to urinary dipsticks that detect glucose concentrations above ~ 3–5 mmol/L [24], automated chemistry analysers with application for urine samples can often quantify urinary concentrations of glucose (uGlu) down to 0.1 mmol/l [25]. Measurement of uCysC, uNAG, and uGlu can be performed with automated chemistry analysers already in use at larger animal healthcare facilities, making measurements practical for clinicians.

The primary aim of this study was to evaluate the potential value of uCysC, uNAG, and uGlu for detection of renal tubular injury by comparing these biomarkers, normalised to urinary creatinine concentrations (uCr) among four groups of dogs: healthy controls, dogs envenomated by *Vipera berus*, and critically ill dogs with or without clinically apparent AKI. The secondary aim was to evaluate these biomarkers over two weeks post-hospitalisation.

## Methods

This prospective observational longitudinal study was performed in Sweden at AniCura Animal Hospital, Stockholm, and at the University of Agricultural Sciences (SLU), Uppsala between March and October 2023. Ethical approval was obtained (5.8.18–09149-2022), and all owners provided written informed consent.

### Animals and study design

Dogs > 6 months of age and of any breed or sex hospitalised for *Vipera berus*-envenomation or intensive care, and healthy dogs, were included. Dogs with known CKD were excluded. The healthy control group (HC) consisted of privately owned dogs deemed healthy by their owners and without abnormal clinical or laboratory findings. Hospitalised dogs were divided into three groups: dogs with clinically apparent AKI (AKI_APP_), dogs envenomated by *Vipera berus* (EVB), and dogs admitted for intensive care without AKI_APP_ (IC). Dogs were assigned to the AKI_APP_ group if diagnosed with AKI during hospitalisation by the attending veterinarian based on historical, clinical, laboratory and imaging information.

On the first day of hospitalisation (day 1), blood was collected during intravenous (IV) catheter placement for hematology and biochemistry analyses. On day 2, blood was drawn from the IV catheter. Urine was collected within 6 h of serum sampling on days 1 and 2. Additional urine samples on days 7 and 14 (± 2 days), were brought to the hospital at the recheck or collected at home and mailed to the laboratory. Sterile urine collection devices were provided, and owners were instructed to collect midstream voided urine, to pipette urine into provided tubes without additive, and to send these by mail on the day of collection. Healthy control dogs were examined and sampled only once (day 1). Medical decisions regarding all hospitalised animals (including euthanasia) were taken by the attending veterinarians and owners, and no medical decisions were influenced by this study. Euthanasia was performed using animal hospital routine (sedation [if needed]: dexmedetomidine 6ug/kg subcutaneously, induction: propofol ~ 2.5 mg/kg IV, and pentobarbital ~ 100 mg/kg IV).

### Laboratory analyses

Serum and urine samples were analysed with Beckman Coulter DxC (Beckman Coulter, Inc, Brea, CA, US). Urine sediment, USG and dipstick were analysed on newly collected urine on day 1 in all dogs. Aliquots of centrifuged urine were stored at −80 °C for batch analysis of creatinine, CysC, Glu, NAG, and protein, and results were normalised to uCr.

Analyses in serum on day 1 (complete blood count, albumin, ALP, ALT, bile acids, calcium, chloride, creatinine, CRP, glucose, phosphate, potassium, sodium, total protein and urea) were analysed immediately. Remaining serum was stored at −80 °C. Additional serum analytes for day 1 (CysC), day 2 (creatinine, CRP, CysC) and all urinary biomarkers were batch analysed within 14 months. Standard reagents for urine samples were used, except for uCysC and uNAG (Additional file 1). Urinary CysC was analysed with an immunoturbidometric method (Gentian Diagnostics, Moss, Norway). Each urine sample was analysed both with the standard method for serum samples and with a modified application (hsCysC) developed in collaboration with Beckman Coulter, with diluted calibrators and increased sample volume to extend measuring range down to 0.03 mg/L. For samples with uCysC concentrations below 0.6 mg/L with the standard method, results from hsCysC were reported. For calculation of ratios, identical units were used, and results were reported without units except for uNAG/uCr for which results are presented as U/g (units/gram).

### Statistical analyses

Statistical calculations were performed using JMP 19 (SAS Institute, Cary, North Carolina). Urinary variables were non-normally distributed and presented as medians and interquartile ranges (IQRs). Age, bodyweight (BW), sex, and urinary biomarkers (uCysC, uNAG, and uGlu) normalised to uCr on days 1 and 2 were compared among groups using the Kruskal–Wallis test. When significant differences were found, Wilcoxon Each Pair tests were used for pairwise comparisons. Significance was set at *p* < 0.05 with Bonferroni correction applied for multiple comparisons. Urinary biomarkers, UPC, sCr, sCysC and serum CRP on day 1 were compared between survivors and non-survivors in the AKI_APP_ group.

## Results

**Fig. 1:**
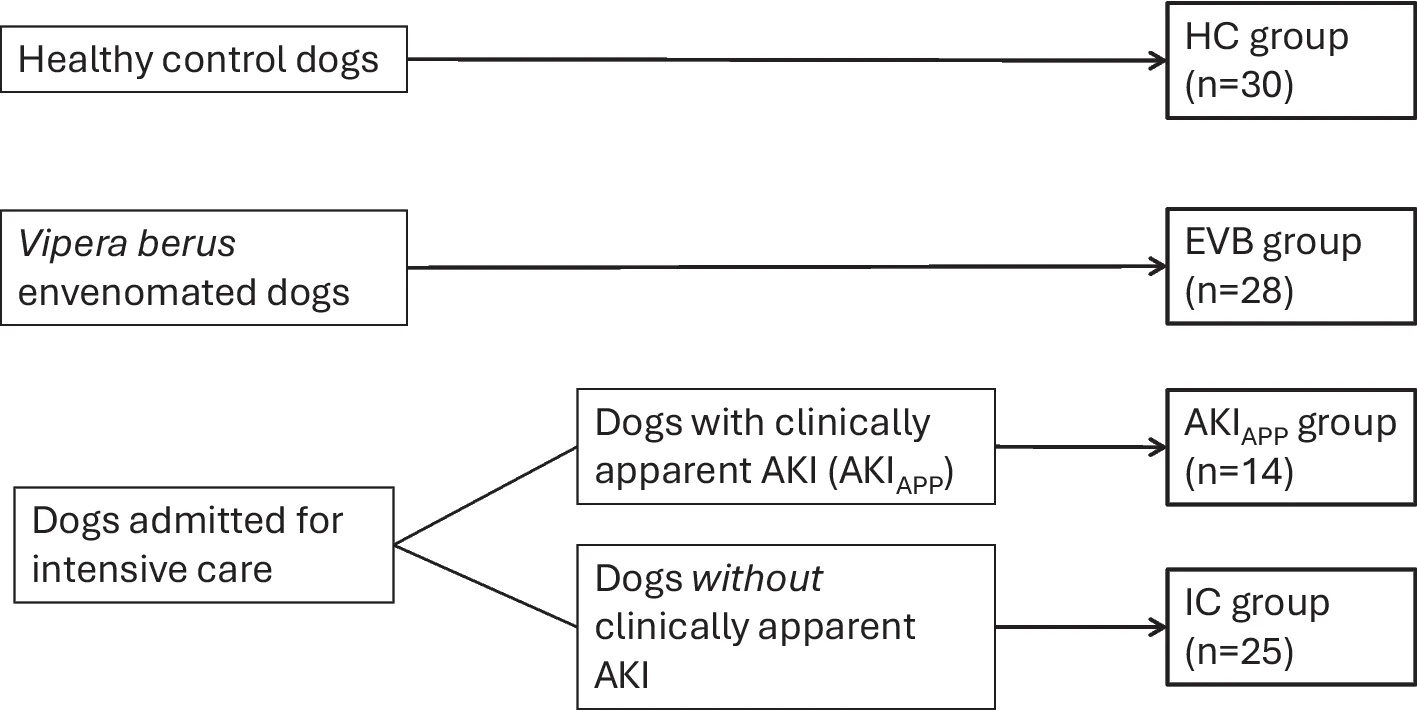
Flow chart of included dogs

A total of 97 dogs were included. The breeds represented were mixed breed (*n* = 8), Labrador retriever (*n* = 7), miniature Schnauzer (*n* = 6), Papillion (*n* = 5), whippet (*n* = 4), and ≤ 3 individuals of 41 other breeds. Median (IQR) age of all dogs was 5.0 (2.1–8.0) years, and median BW was 14.6 (9.8–26.5) kg. There were 53 females of which 29 were spayed, and 44 males of which 26 were neutered. There were 30 healthy control dogs, 28 dogs envenomated by *Vipera berus* and 39 dogs admitted for intensive care for a variety of reasons, 14 of which were diagnosed with AKI by the attending clinician and assigned to the AKI_APP_ group, and 25 that were not given a clinical diagnosis of AKI (IC group). A flow chart describing the inclusion process is provided in Fig. 1). On day 1, 3 of the dogs with AKI_APP_ were in IRIS (international renal interest society) grade 1, 1 dog in grade 2, 6 dogs in grade 3 and 4 dogs in grade 4 AKI. The reasons for AKI in the AKI_APP_ dogs were acute glomerulopathy (*n* = 4), leptospirosis (*n* = 2), previous anaesthesia/sedation (*n* = 2), vitamin D intoxication (*n* = 1), ibuprofen intoxication (*n* = 1), pyelonephritis (*n* = 1), and unknown (*n* = 3). The 25 dogs in the IC group were diagnosed with acute gastroenteritis (*n* = 9), pyometra (*n* = 4), pancreatitis (*n* = 3), xylitol intoxication (*n* = 1), ethylene glycol ingestion without AKI_APP_ (*n* = 1), liver failure (*n* = 2), congestive heart failure (CHF; *n* = 2), gastric dilatation volvulus (*n* = 1), DIC for unknown reason (*n* = 1), and postoperative atrioventricular block (*n* = 1). No *Vipera berus*-envenomated dogs were diagnosed with AKI by the attending clinician. The elapsed time between snake bite and inclusion in the EVB group was 3–24 h, and the duration of clinical signs before inclusion in dogs in the IC group was 2–72 h. Length of hospitalisation was 2–7 days, depending on how the individual dog responded to treatment. In all dogs except healthy controls and the two dogs with CHF, fluid therapy was initiated before urine samples were collected. Treatment of individual dogs was not influenced by this study.

Day 1 urine was collected by cystocentesis in 11/14 dogs with AKI_APP_ and in 2/25 dogs in the IC group. All other samples were spontaneously voided. One hundred out of 192 samples had uCysC concentrations below 0.6 mg/L with the standard method, and results from a high-sensitivity method (hsCysC) were reported. Results below the measuring range for hsCysC (< 0.03 mg/L, *n* = 50/192), uNAG (< 1.6 U/L, *n* = 21/192), and uGlu (< 0.03 mmol/L, *n* = 3/192) were set to half this value.

**Fig. 2:**
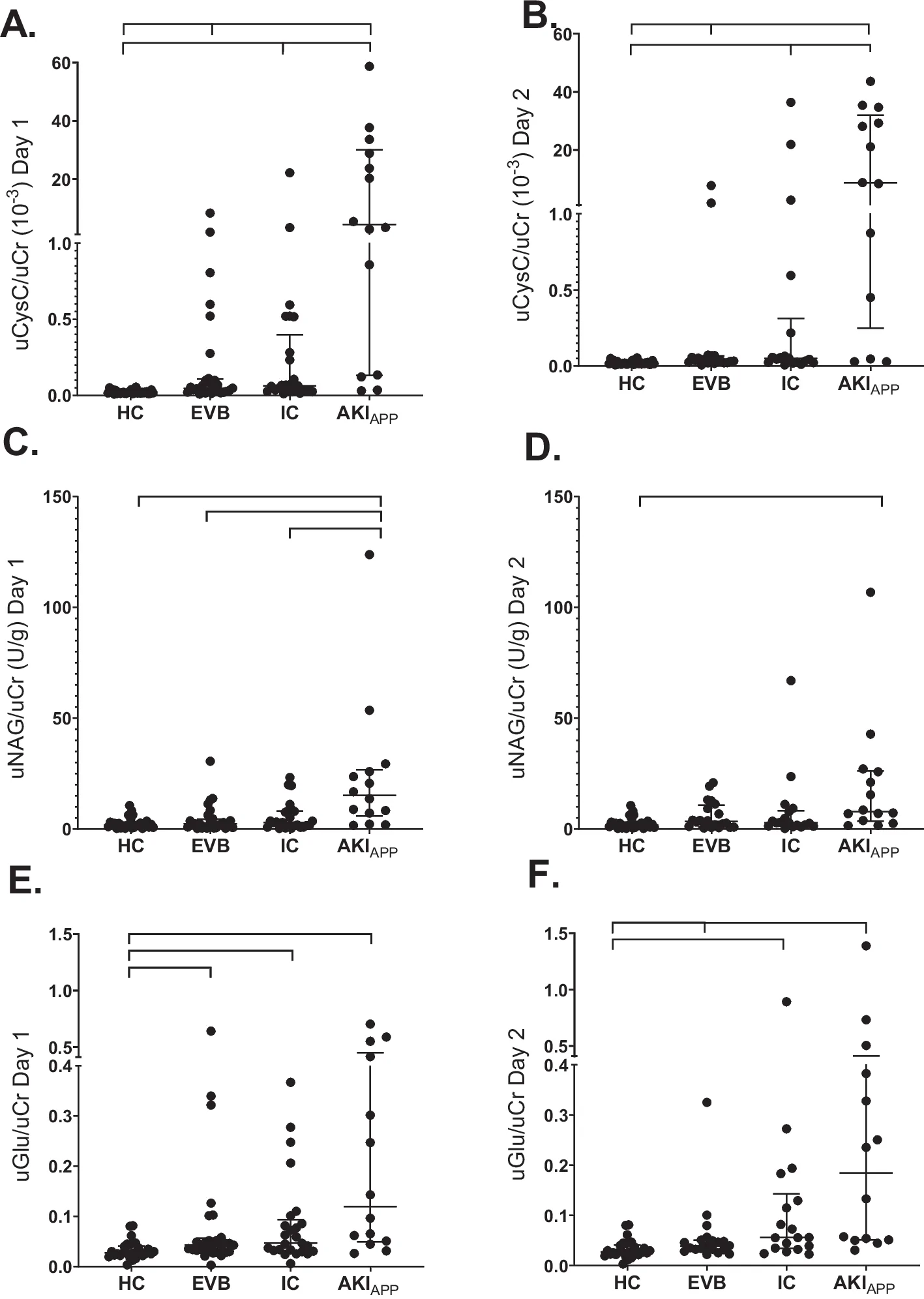
Urinary CysC/uCr (**A**-**B**), uNAG/uCr (**C**-**D**) and uGlu/uCr (**E**–**F**) on day 1 (left) and day 2 (right) in healthy control dogs (HC), dogs envenomated by *Vipera berus* (EVB), dogs admitted for intensive care for a variety of reasons (IC), and dogs with clinically apparent acute kidney injury (AKI_APP_). Significant differences (*p* < 0.008) are marked with horizontal bars above the figures. Vertical bars within the figures represent median and interquartile range. uCysC: urinary cystatin C, uCr: urinary creatinine, uGlu: urinary glucose, uNAG: urinary *N*-acetyl-β-D-glucosaminidase

There were no differences in age, sex, or BW among the 4 groups of dogs. Demographical and clinicopathological data of included dogs are presented in Table 1. The highest values of each urinary marker in the healthy control group are shown, as concentrations and normalised to creatinine, in Table 2. On day 1, compared to the HC group (median 0.02 [IQR 0.01–0.03]), uCysC/uCr was higher in AKI_APP_ (4.4 [0.13–30.0], *p* < 0.0001), EVB (0.05 [0.03–0.11], *p* < 0.0001), and IC dogs (0.06 [0.03–0.40], *p* < 0.0001; Fig. 2). Urinary Glu/uCr on day 1 was also higher in AKI_APP_ (median 0.12, [0.05–0.45], *p* < 0.0001), EVB (0.04 [0.03–0.06], *p* < 0.004), IC (0.05 [0.03–0.09], *p* < 0.0008) than in the HC group (0.03 [0.02–0.04], Fig. 2). Urinary concentrations of the 3 biomarkers in all groups of dogs are shown in Additional file 2.

**Table 1:** Demographics and clinicopathological variables (median and IQR) for included dogs on day 1

	HC (*n* = 30)	EVB (*n* = 28)	IC (*n* = 25)	AKI_APP_ (*n* = 14)
Age (years)	4.3 (1.5–6.6)^a^	5.0 (2.0–8.8)^a^	5.0 (2.0–9.0)^a^	5.6 (3.8–10.3)^a^
Weight (kg)	20.5 (9.9–29.8)^a^	15.2 (11.0–27.0)^a^	10.3 (6.1–24.5)^a^	14.4 (8.8–20.3)^a^
Sex (F/FC/M/MC)	13/6/6/5^a^	11/0/13/4^a^	14/2/9/0^a^	6/0/6/2^a^
uCrea (umol/L)	18,962 (10,485–25784)^a^	11,712 (6381–18,104)^ab^	7574 (3580–14334)^b^	4492 (3710–9280)^b^
USG	1.046 (1.033–1.051)	1.028 (1.017–1.042)	1.020 (1.013–1.036)	1.014 (1.013–1.017)
UPC	0.11 (0.09–0.14)	0.22 (0.16–0.44)	0.33 (0.16–1.28)	6.0 (0.65–11.3)
sCrea (umol/L)	78.7 (66.5–87.1)	77.8 (62.0–88.6)	67.8 (60.1–78.2)	321.9 (194.1–488.6)
sCysC (mg/L)	0.50 (0.38–0.73)	0.49 (0.34–0.59)	0.42 (0.33–0.65)	2.0 (1.1–3.5))
CRP	4.4 (3.6–6.7)	6.7 (5.1–46.2)	79.0 (19.8–217.8)	60.4 (15.1–109.0)

*AKI*_*APP*_ Apparent acute kidney injury, *HC* Healthy control dogs, *EVB* Dogs envenomated by *Vipera berus, IC* Dogs admitted for intensive care, without clinically apparent AKI, *AKI*_*APP*_ Dogs with clinically apparent acute kidney injury, *IQR* Interquartile range, *uCrea* Urinary creatinine, *F* Female, *FC* Female castrated, *M* Male, *MC* Male castrated, *USG* Urine specific gravity, *UPC* Urine protein to creatinine ratio, *sCrea* Serum creatinine, *sCysC* Serum cystatin C concentration, *CRP* C-reactive protein

Significant differences (*p* < 0.008) are noted where superscripted letters differ among groups. For USG, UPC, sCrea, sCysC and CRP, comparisons among groups were not performed because these variables were used for group affiliation

**Table 2:** Highest value for each urinary marker in urine from 30 healthy control dogs on day 1

	Urinary concentration	Normalised to creatinine
uCysC	0.1 mg/L	0.05 × 10^–3^
uNAG	17.5 U/L	10.6 U/g
uGlu	1.5 mmol/L	0.08

*uCysC* Urinary cystatin C, *uNAG* Urinary *N*-acetyl-β-D-glucosaminidase, *uGlu* Urinary glucose

**Fig. 3:**
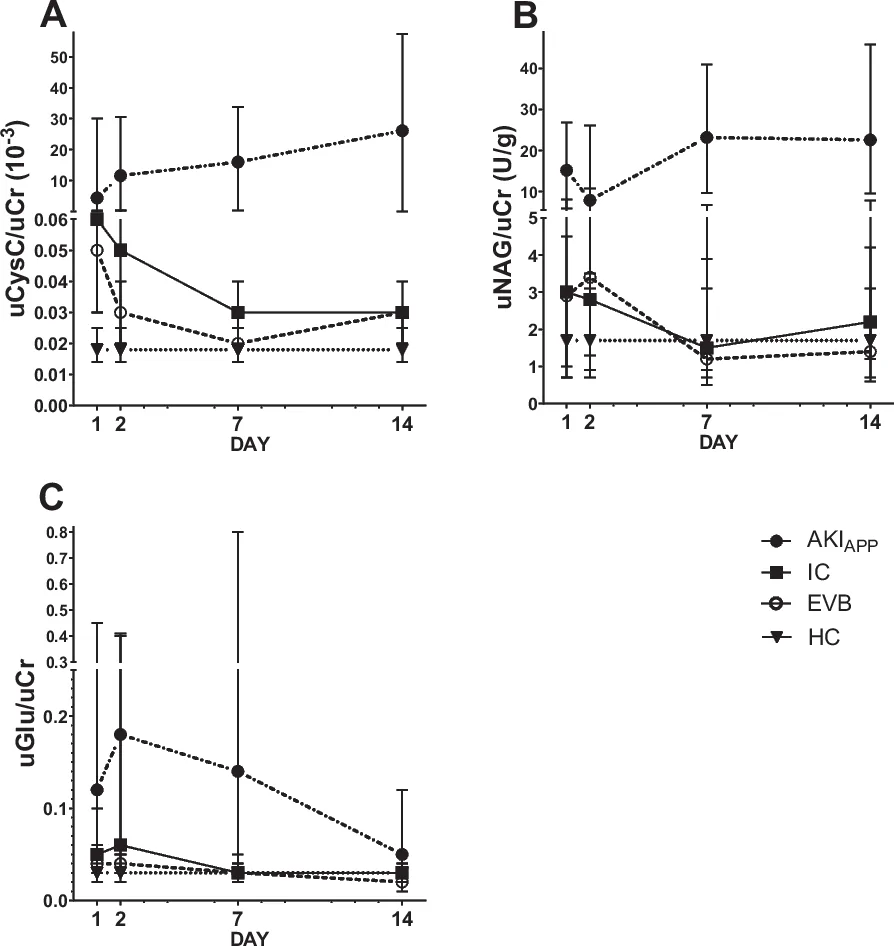
Median urinary (**A**) CysC/uCr, (**B**) uNAG/uCr, and (**C**) uGlu/uCr in dogs with clinically apparent acute kidney injury (AKI_APP_), dogs admitted for intensive care (IC), *Vipera berus-* envenomated dogs (EVB), and healthy control dogs (HC) sampled at days 1, 2, 7, and 14. Vertical bars represent interquartile ranges. Note that healthy dogs were only sampled on day 1

In the AKI_APP_ group, on day 1 (and on day 2), 12 of 14 (86%) dogs had a uCysC/uCr above the highest value of the control dogs. The corresponding numbers for uNAG/uCr, and uGlu/uCr on day 1 was 8 of 14 (57%). On day 2, uNAG/uCr was increased in 6/14 (43%) dogs, and uGlu/uCr in 8 of 14 (57%) dogs (Fig. 2). All urinary markers decreased over two weeks post hospitalisation in all groups except AKI_APP_. Figure 3 shows urinary biomarkers, normalised to creatinine, over two weeks post hospitalisation. Additional file 3 shows serum concentrations of CysC and uCysC/uCr, respectively, for all individual dogs on day 1. The number of samples available from dogs in the AKI_APP_ group were 10 on day 7 and 5 on day 14. Corresponding numbers for the IC group were 8 on day 7, and 4 on day 14, and for the EVB group 12 on day 7, and 5 on day 14. Twenty-seven of the 44 samples from day 7 and 14 were obtained in hospital and 17 were sent by mail. Sixteen of the 17 samples sent by mail were frozen within 30 h and one sample was frozen within 48 h.

**Fig. 4:**
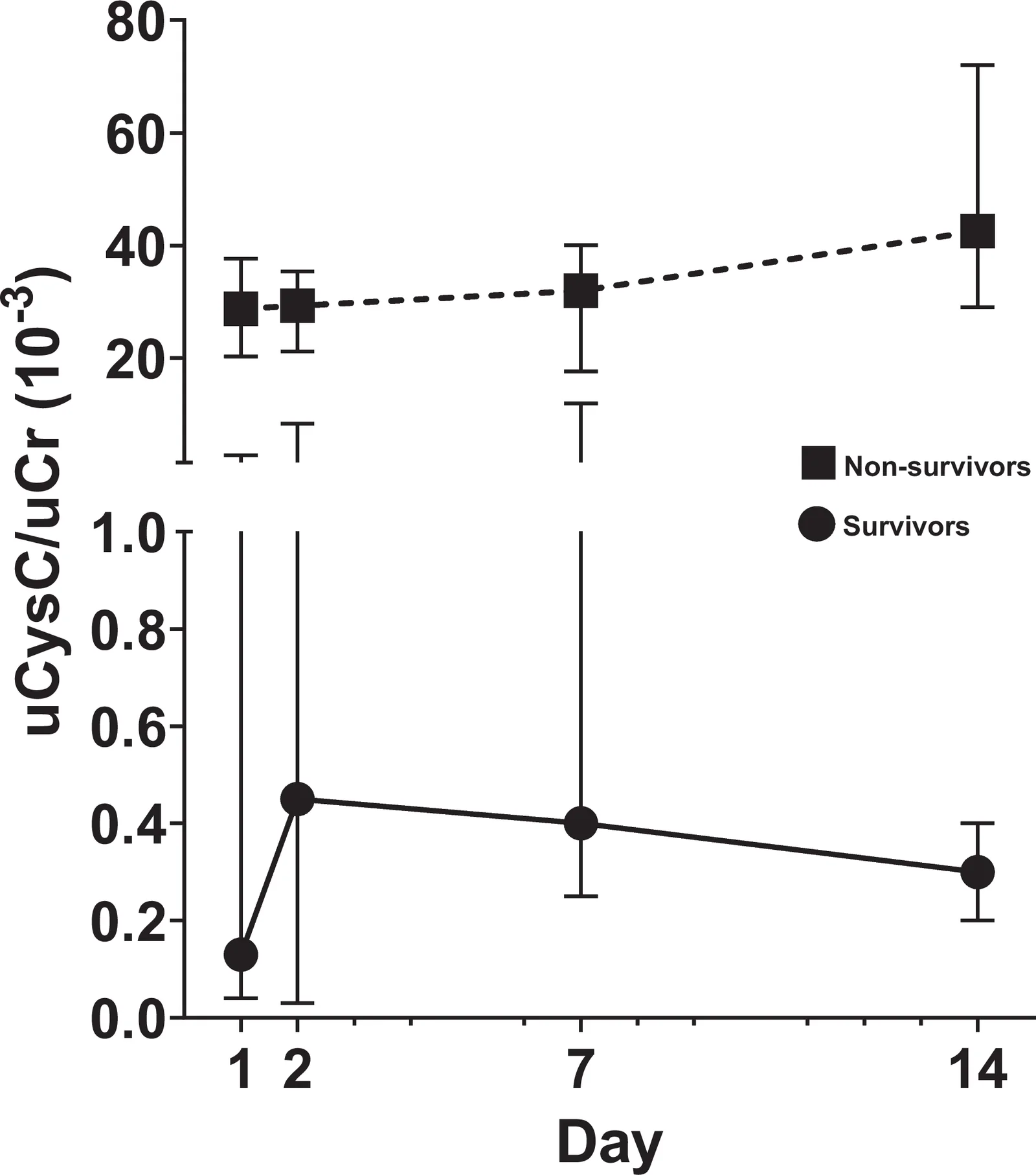
Median urinary CysC/uCr in survivors and non-survivors in the group of dogs with clinically apparent acute kidney injury, over time. Vertical bars represent interquartile ranges

Seven of the 14 dogs in the AKI_APP_ group survived > 6 months post hospitalisation, and 7 dogs died or were euthanised during the first 2 weeks (*n* = 4) or 2 months (*n* = 3) post hospitalisation. The 7 dogs with AKI_APP_ that did not survive had a significantly (*p* < 0.002) higher uCysC/uCr (median 28.8, IQR 20.3–37.7) on day 1, than the 7 dogs with AKI_APP_ that survived (0.13 [0.04–2.8], Fig. 4). This was also true for uCysC (not normalised), sCysC, but not for sCr, UPC, uNAG/uCr or uGlu/uCr (Table 3). Furthermore, uCysC/uCr remained stable or increased during follow-up period in the dogs that died, while the dogs that survived had values of uCysC that decreased (Fig. 4).

**Table 3:** Urinary and serum analytes in dogs with clinically apparent acute kidney injury on day 1

	AKI_APP_ Survivors (median, IQR) (*n* = 7)	AKI_APP_ Non-Survivors (median, IQR) (*n* = 7)	*p*-value
uCysC/uCr (10^–3^)	0.13 (0.04–2.8)	28.8 (20.3–37.7)	0.002*
uCysC (mg/L)	0.08 (0.01–4.1)	11.1 (9.1–23.1)	0.002*
uNAG/uCr (U/g)	8.8 (1.9–26.0)	20.6 (8.3–53.5)	0.25
uGlu/uCr	0.05 (0.03–0.14)	0.3 (0.1–0.6)	0.07
UPC	0.8 (0.14–7.3)	4.9 (1.0–13.4)	0.06
sCrea (µmol/L)	214 (113–343)	412 (278–492)	0.07
sCysC (mg/L)	1.2 (0.7–1.3)	3.5 (2.4–4.6)	0.002*
sCRP (mg/L)	46 (14.7–109-4)	74.9 (15–96.4)	0.8

*AKI*_*APP*_ Apparent acute kidney injury, *IQR* Inter quartile range, *uCysC* Urinary cystatin C, *uCrea* Urinary creatinine, *uNAG* Urinary *N*-acetyl-β-D-glucosaminidase, *uGlu* Urinary glucose, *UPC* Urinary protein to creatinine ratio, *sCrea* Serum creatinine concentration, *sCysC* Serum cystatin C concentration, *sCRP* Serum C-reactive protein concentration

^*^Significant difference (*p* < 0.05)

In the group of dogs envenomated by *Vipera berus*, 12 of 28 (44%) dogs had an uCysC/uCr above the highest value of the control dogs on day 1. The corresponding number for uNAG/uCr was 4 of 28 (14%), and for uGlu/uCr 6 of 28 (21%). No dog in the EVB group died or was euthanised. In the IC group, 16 of 25 dogs (56%) had a uCysC/uCr above the highest value of the healthy dogs on day 1. The corresponding number for uGlu/uCr was 9 of 25 (36%), and for uNAG/uCr 5 of 25 (20%). Three dogs in the IC group died (*n* = 1) or were euthanised during hospitalisation because of lack of response to treatment (*n* = 2). All 3 dogs had increased uCysC/uCr.

## Discussion

This study compared urinary CysC, NAG, and Glu, normalised to uCr, over 14 days in dogs envenomated by *Vipera berus* and in critically ill dogs with and without clinically apparent AKI. On day 1 and 2, uCysC/uCr and uGlu/uCr were significantly higher in all groups of hospitalised dogs than in the healthy group. Several studies have shown an association between experimentally induced critical illness (hypotension, toxicity) and spontaneous AKI and increases in urinary markers of renal injury [14, 15, 26–30]. An increased value of any biomarker (which was the case in 95/192 samples in the present study) was accompanied by an increased value in 1–2 of the other markers in 62% (59/95) of samples. This indicates that renal tubular injury or dysfunction was prevalent in this population of hospitalised dogs. A definitive diagnosis of tubular injury would have required renal biopsies, but this was not feasible in this observational study because the decision to perform renal biopsy was not taken in any of the included dogs. The association between histopathological evidence of tubular injury and increased uCysC/uCr, uNAG/uCr and uGlu/uCr has been shown previously in dogs [14–16]. Tubular dysfunction however, might not even be evident on histological examination of renal tissue, as illustrated in a study on canine Fanconi syndrome [31], making confirmation of its presence even more difficult. The absence of a gold standard poses a significant challenge when evaluating the diagnostic accuracy of tubular injury markers. The use of multiple urinary markers likely increases diagnostic accuracy and should be considered, especially in clinical situations.

In the present study, uCysC/uCr was increased in 44% of EVB dogs. This is in accordance with earlier studies which have described elevations in several other urinary biomarkers in *Vipera berus*-envenomated dogs, indicating that transient renal tubular injury is prevalent even in the absence of azotaemia [32, 33]. In the study by Harjen et al*.*, uCysC/uCr was not higher in snake bitten dogs than in healthy controls [32]. This may have been related to the choice of analytic method. In one study of six experimentally hypotensive greyhounds [26], a multiplex bead-based assay, such as the one used by Harjen et al*.*, showed lower uCysC concentrations than a canine CysC ELISA and a PETIA, both validated for canine urine [12, 34]. This discrepancy may have been caused by poor cross-reactivity and emphasises the importance of method validation. Urinary CysC concentrations in the healthy dogs were low, most often below 0.1 mg/L, while some dogs with AKI_APP_ had urinary concentrations above 30 mg/L. This represents a major methodological challenge. To detect low concentrations and avoid problems with antigen excess in samples with high uCysC content, urine samples in the present study were analysed both with the standard serum method and with a high-sensitivity method. While the standard method is likely adequate for most clinically relevant elevations, it may not detect small elevations of uCysC in dilute urine.

Over 2 weeks post hospitalisation, uCysC/uCr gradually decreased in all groups except AKI_APP_. On day 14, almost all EVB and IC dogs that survived had values approaching the range of healthy dogs. Among the 14 AKI_APP_ dogs, seven survived beyond six months while seven died or were euthanised within two months. Non-survivors had higher uCysC/uCr on day 1 (and day 2) than survivors. This was also true for both serum and urinary concentrations of CysC but not for the routinely used markers sCrea and UPC. Furthermore, uCysC/uCr remained high or increased in the dogs that died or were euthanised and decreased in survivors over two weeks post hospitalisation. These results indicate that uCysC, normalised or not, could potentially be of value as a prognostic marker, but further prospective studies are needed to confirm this hypothesis.

Because CysC is freely filtered through the glomeruli and reabsorbed by proximal tubular cells [10, 11], an increased concentration of CysC in serum could potentially result in an increased uCysC concentration. Twenty-six of the 40 dogs with increased uCysC/uCr in the present study had a serum CysC within the range of the healthy control dogs (< 0.9 mg/L), indicating that tubular injury or dysfunction was the probable reason for increased uCysC/Cr in many dogs. Urinary biomarkers of renal injury and dysfunction are, however, particularly warranted for detection of kidney injury prior to the onset of azotaemia, and in those situations, serum CysC concentrations are usually not increased. Another factor to consider when interpreting uCysC is glomerular proteinuria. Urinary albumin and CysC compete for the same proximal tubular cell receptors [35], and with glomerular range proteinuria (UPC > 3), inhibition of the uCysC reabsorption might occur, resulting in increased uCysC. In the present study, 8 dogs had a UPC > 3 and therefore, UPC receptor competition possibly had some impact on study results.

There were 23 dogs with uGlu/uCr above the highest value of the control dogs and only 2 of these were also detected on dipsticks, meaning that for 21 dogs, uGlu/uCr added new information. This is in accordance with results of a Swiss study that described uGlu as a sensitive laboratory marker for discrimination of AKI from CKD in dogs [36]. The lowest uGlu concentration detected on the dipstick in the present study was 3.4 mmol/l. Urinary NAG/uCr was higher in the AKI_APP_ group than in the other three groups, but there were no differences among other groups. Therefore, uNAG/uCr was considered potentially less valuable than uCysC/uCr and uGlu/uCr as markers of renal tubular injury in this study.

Urinary markers were mainly presented normalised to uCr in the present study. In people, the use of urinary creatinine for normalisation of urinary markers in AKI is controversial [37], and the situation is probably similar for dogs. However, the use of urinary concentration of biomarkers also has its limitations because of the varying urine dilution in clinical AKI patients. All dogs except the healthy controls and the two dogs in congestive heart failure were treated with IV fluids before urine samples were obtained, further contributing to the decision to normalise urinary biomarkers. As shown in Additional file 2, results for urinary concentrations of biomarkers show similar patterns as those for normalised markers, shown in Fig. 2. Also, the comparison of uCysC between AKI survivors and non-survivors yielded similar results for normalised and non-normalised values.

This study had some limitations. Because of noncompliance on the part of owners, and because several dogs did not survive to discharge, the number of urine samples on day 7 and 14 were low, resulting in loss of longitudinal information. Also, the decision to have owners send urine samples by mail from day 7 and 14 resulted in samples being kept in room temperature for up to 48 h, which might have affected results.

## Conclusions

Urinary CysC/uCr and uGlu/uCr were markedly increased in dogs with clinically apparent AKI and they were also increased in several critically ill and snake-bitten dogs, suggesting presence of renal tubular injury. These markers, analysed with common biochemistry instruments, have potential use for detection of tubular injury or dysfunction in dogs, preferably as part of a diagnostic panel of urinary biomarkers.

## Supplementary Information

Additional file 1: Table showing reagents used for included urinary analytes, and CV% for samples with low and high concentrations, as well as linearity upon dilution (Observed to Expected (O/E) ratio), analysed with the instrument Beckman Coulter DxC.

Additional file 2: Figure showing urinary concentrations of biomarkers on day 1 in all groups of dogs.

Additional file 3: Table showing serum concentrations of cystatin C and uCysC/uCr results individually for all included dogs.

## Data Availability

The dataset generated and analysed in this study is available from the corresponding author on reasonable request.

## Abbreviations

AKI: Acute kidney injury
AKI_APP_: Clinically apparent acute kidney injury
HC: Control group
CKD: Chronic kidney disease
Crea: Creatinine
CRP: C-reactive protein
CysC: Cystatin C
EVB: Group of dogs envenomated by *Vipera* *berus*
FE: Fractional excretion
GFR: Glomerular filtration rate
Glu: Glucose
hsCysC: Cystatin C analysed with the high-sensitivity method
IC: Group of dogs admitted for intensive care, without clinically apparent acute kidney injury
NAG: *N*-Acetyl-β-D-glucosaminidase
sCysC: Serum cystatin C
uCr: Urinary creatinine
uCysC: Urinary cystatin C
UPC: Urine protein to creatinine ratio
uProt: Urinary protein
