# Supplementary material for: Evaluation of urinary biomarkers for detection of renal tubular injury in Vipera berus-envenomated and critically ill dogs with and without clinically apparent acute kidney injury
Source: BMC Vet Res. 2026 Sep 21;22:546. doi: 10.1186/s12917-026-05939-1 (PMC13595791; doi:10.1186/s12917-026-05939-1)
Supplement: Supplementary file 1 [file 12917_2026_5939_MOESM1_ESM.pdf]

Additional file 1. Reagents used for included analytes, and CV% for samples with low and high concentrations, as well as linearity upon dilution (Observed to Expected (O/E) ratio) for included urinary analytes, analysed with the instrument Beckman Coulter DxC.

	**Method and reagent producer**	**CV %, low conc (mean)**	**CV %, high conc (mean)**	**O/E % (dilutional range)**
**uCysC**	Immunoturbidometric, Gentian Diagnostics	2.1 (0.49 mg/L)	0.5 (5.5 mg/L)	92-102 (0.5-10.2 mg/L)
**hsCysC**	Immunoturbidometric, Gentian Diagnostics	3.5 (0.03 mg/L)	2.0 (0.57 mg/L)	81-120 (0.03-0.6 mg/L)
**uCrea**	Enzymatic, Beckman Coulter	1.2 (506 μmol/L)	0.8 (28259 μmol/L)	94-104 (886-24383 μmol/L)
**uGlu**	Hexokinase/G-6-PHD, Beckman C	13.9 (0.03 mmol/L)	0.9 (8.4 mmol/L)	96-106 (0.03-3.4 mmol/L)
**uNAG**	Colorimetric, Diazyme	2.8 (2.0 U/L)	0.7 (18.0 U/L)	100-112 (1.7-94.9 U/L)
**uProt**	Pyrogallo red, Beckman Coulter	7.3 (0.06 g/L)	0.8 (0.7 g/L)	87-103 (0.03-1.6 g/L)

CV: coefficient of variation, conc: concentration, IFCC: International federation of clinical chemistry, uCrea: urinary creatinine concentration, uCysC; urinary cystatin C concentration, uGlu, urinary glucose concentration, hsCysC: high sensitivity cystatine C concentration, uNAG: urinary N-acetyl-β-D-glucosaminidase concentration, uProt: urinary protein concentration.
