# Supplementary material for: Evaluation of urinary biomarkers for detection of renal tubular injury in Vipera berus-envenomated and critically ill dogs with and without clinically apparent acute kidney injury
Source: BMC Vet Res. 2026 Sep 21;22:546. doi: 10.1186/s12917-026-05939-1 (PMC13595791; doi:10.1186/s12917-026-05939-1)

Urinary concentrations of biomarkers day 1

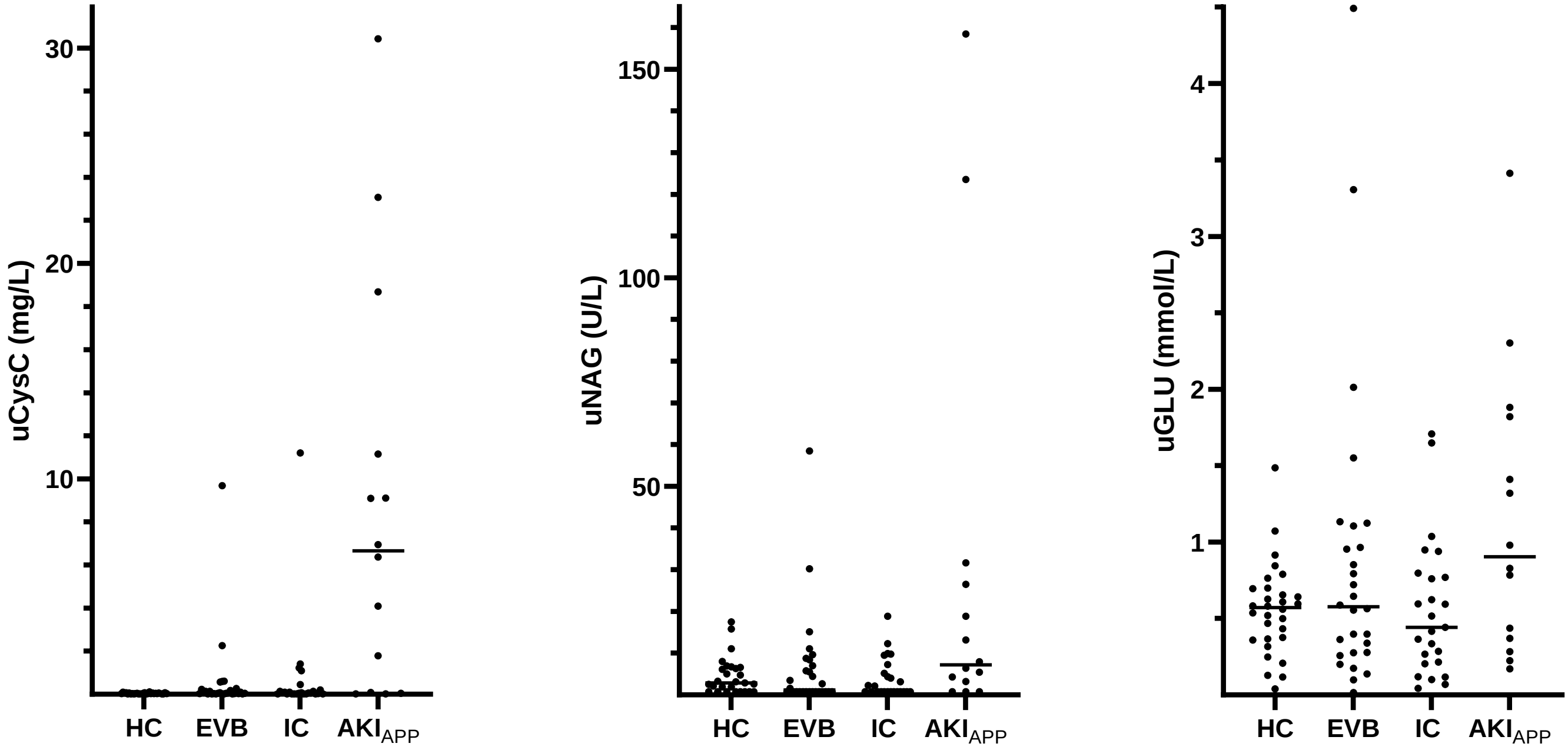

Supplement: Supplementary file 2 [file 12917_2026_5939_MOESM2_ESM.pdf]
