# Supplementary material for: Evaluation of urinary biomarkers for detection of renal tubular injury in Vipera berus-envenomated and critically ill dogs with and without clinically apparent acute kidney injury
Source: BMC Vet Res. 2026 Sep 21;22:546. doi: 10.1186/s12917-026-05939-1 (PMC13595791; doi:10.1186/s12917-026-05939-1)
Supplement: Supplementary file 3 [file 12917_2026_5939_MOESM3_ESM.pdf]

Additional file 2. Serum concentrations of cystatin C and urinary cystatin C/urinary creatinine ratio in all included dogs on day 1. Bold numbers in the sCysC column represent numbers higher than the range of values in the healthy control dogs for serum cystatin C concentration (<0.9 mg/L). Similarly, bold numbers in the uCysC/uCr column represent numbers higher than the range of values in the healthy control dogs (< 0.05 x 10^{-3}).

ID	sCysC (mg/L)	uCysC/uCr
AKI 1	**3.14**	**28.8 x 10^{-3}**
AKI 2	**2.44**	**33.6 x 10^{-3}**
AKI 3	**3.60**	**23.7 x 10^{-3}**
AKI 4	**2.21**	**58.7 x 10^{-3}**
AKI 5	**3.52**	**20.3 x 10^{-3}**
AKI 6	**4.58**	**37.7 x 10^{-3}**
AKI 7	**1.9**	0.03 x 10^{-3}
AKI 8	**1.2**	**3.38 x 10^{-3}**
AKI 9	0.71	**0.12 x 10^{-3}**
AKI 10	**1.31**	0.03 x 10^{-3}
AKI 11	0.77	**0.86 x 10^{-3}**
AKI 12	**4.65**	**5.43 x 10^{-3}**
AKI 13	0.48	**0.13 x 10^{-3}**
AKI 14	**1.21**	**2.79 x 10^{-3}**
EVB 1	0.50	0.04 x 10^{-3}
EVB 2	**3.80**	0.02 x 10^{-3}
EVB 3	**2.38**	**0.81 x 10^{-3}**
EVB 4	0.38	0.02 x 10^{-3}
EVB 5	0.26	0.05 x 10^{-3}
EVB 6	0.34	0.03 x 10^{-3}
EVB 7	0.42	**0.07 x 10^{-3}**
EVB 8	0.59	**0.10 x 10^{-3}**
EVB 9	0.34	**0.11 x 10^{-3}**
EVB 10	0.29	0.03 x 10^{-3}
EVB 11	0.58	**0.60 x 10^{-3}**
EVB 12	0.34	**1.81 x 10^{-3}**
EVB 13	0.35	**0.10 x 10^{-3}**
EVB 14	0.41	0.04 x 10^{-3}
EVB 15	**1.41**	**8.34 x 10^{-3}**
EVB 16	NA	0.04 x 10^{-3}
EVB 17	NA	**0.07 x 10^{-3}**
EVB 18	0.33	0.04 x 10^{-3}
EVB 19	NA	**0.52 x 10^{-3}**
EVB 20	0.59	0.03 x 10^{-3}
EVB 21	0.54	0.05 x 10^{-3}
EVB 22	0.51	**0.07 x 10^{-3}**

EVB 23	0.54	0.02×10^{-3}
EVB 24	NA	0.01×10^{-3}
EVB 25	0.48	0.01×10^{-3}
EVB 26	0.62	0.02×10^{-3}
EVB 27	0.54	**0.28×10^{-3}**
EVB 28	0.46	0.02×10^{-3}
IC 1	0.28	**0.05×10^{-3}**
IC 2	0.37	0.03×10^{-3}
IC 3	0.42	0.01×10^{-3}
IC 4	0.38	0.04×10^{-3}
IC 5	0.69	**0.52×10^{-3}**
IC 6	0.39	**0.28×10^{-3}**
IC 7	0.67	**0.52×10^{-3}**
IC 8	0.41	0.03×10^{-3}
IC 9	0.56	0.02×10^{-3}
IC 10	0.32	**0.06×10^{-3}**
IC 11	0.75	**0.06×10^{-3}**
IC 12	0.40	**0.07×10^{-3}**
IC 13	0.28	**3.36×10^{-3}**
IC 14	0.89	**0.23×10^{-3}**
IC 15	0.16	**0.07×10^{-3}**
IC 16	0.51	0.03×10^{-3}
IC 17	NA	**22.18×10^{-3}**
IC 18	0.33	**0.05×10^{-3}**
IC 19	0.29	**0.07×10^{-3}**
IC 20	0.53	0.01×10^{-3}
IC 21	**0.93**	0.03×10^{-3}
IC 22	0.65	**0.11×10^{-3}**
IC 23	NA	0.03×10^{-3}
IC 24	0.61	**0.52×10^{-3}**
IC 25	0.42	**0.59×10^{-3}**
